# Association between weight change and incidence of cardiovascular disease events and mortality among adults with type 2 diabetes: a systematic review of observational studies and behavioural intervention trials

**DOI:** 10.1007/s00125-021-05605-1

**Published:** 2021-12-02

**Authors:** Jean Strelitz, Emma R. Lawlor, Yue Wu, Annabel Estlin, Giri Nandakumar, Amy L. Ahern, Simon J. Griffin

**Affiliations:** 1 MRC Epidemiology Unit, Institute of Metabolic Science, School of Clinical Medicine, University of Cambridge, Cambridge, UK; 2 School of Clinical Medicine, University of Cambridge, Cambridge, UK; 3 Primary Care Unit, Department of Public Health and Primary Care, School of Clinical Medicine, University of Cambridge, Cambridge, UK

**Keywords:** Cardiovascular complications, Cardiovascular disease, Epidemiology, Meta-analysis, Systematic review, Type 2 diabetes, Weight change, Weight loss, Weight management

## Abstract

**Aims/hypothesis:**

Weight loss is often recommended in the treatment of type 2 diabetes. While evidence has shown that large weight loss may lead to diabetes remission and improvement in cardiovascular risk factors, long-term impacts are unclear. We performed a systematic review of studies of weight loss and other weight changes and incidence of CVD among people with type 2 diabetes.

**Methods:**

Observational studies of behavioural (non-surgical and non-pharmaceutical) weight changes and CVD events among adults with type 2 diabetes, and trials of behavioural interventions targeting weight loss, were identified through searches of MEDLINE, EMBASE, Web of Science, CINAHL, and The Cochrane Library (CENTRAL) until 9 July 2019. Included studies reported change in weight and CVD and/or mortality outcomes among adults with type 2 diabetes. We performed a narrative synthesis of observational studies and meta-analysis of trial data.

**Results:**

Of 13,227 identified articles, 17 (14 observational studies, three trials) met inclusion criteria. Weight gain (vs no change) was associated with higher hazard of CVD events (HRs [95% CIs] ranged from 1.13 [1.00, 1.29] to 1.63 [1.11, 2.39]) and all-cause mortality (HRs [95% CIs] ranged from 1.26 [1.12, 1.41] to 1.57 [1.33, 1.85]). Unintentional weight loss (vs no change) was associated with higher risks of all-cause mortality, but associations with intentional weight loss were unclear. Behavioural interventions targeting weight loss showed no effect on CVD events (pooled HR [95% CI] 0.95 [0.71, 1.27]; *I*^2^ = 50.1%). Risk of bias was moderate in most studies and was high in three studies, due to potential uncontrolled confounding and method of weight assessment.

**Conclusions/interpretation:**

Weight gain is associated with increased risks of CVD and mortality, although there is a lack of data supporting behavioural weight-loss interventions for CVD prevention among adults with type 2 diabetes. Long-term follow-up of behavioural intervention studies is needed to understand effects on CVD and mortality and to inform policy concerning weight management advice and support for people with diabetes.

**PROSPERO registration** CRD42019127304.

**Graphical abstract:**

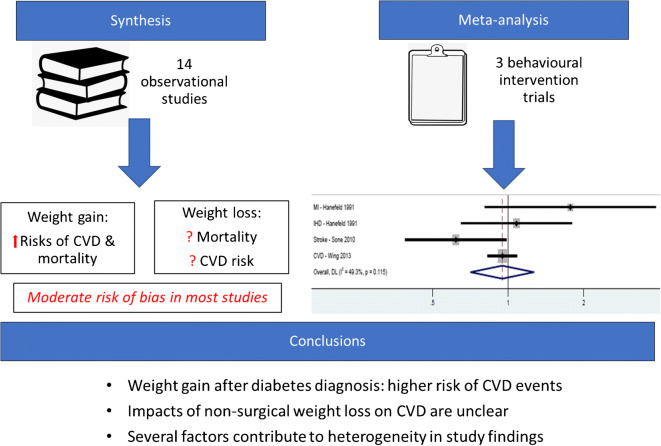

**Supplementary Information:**

The online version contains peer-reviewed but unedited supplementary material available at 10.1007/s00125-021-05605-1.

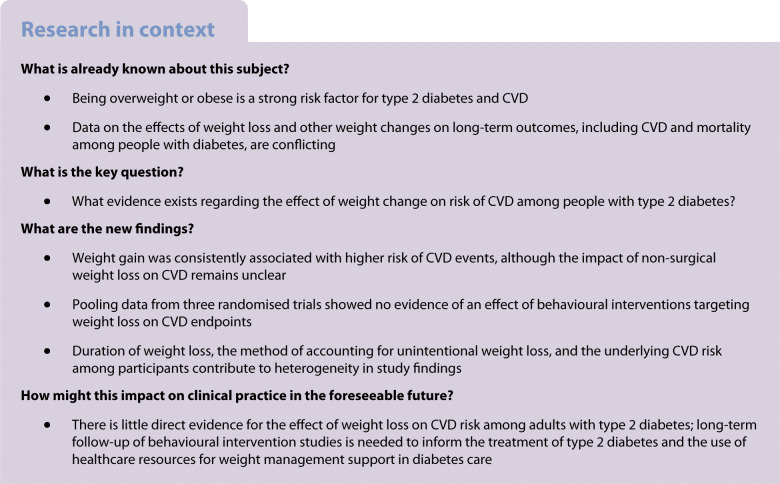



## Introduction

Being overweight or obese is a strong risk factor for type 2 diabetes and CVD. International guidelines recommend that people with type 2 diabetes who are obese or overweight should be encouraged to make behavioural changes to achieve sustained weight loss. Weight loss among overweight or obese people may lead to diabetes remission [1] and improvements in some cardiovascular risk factors [2], although the impact on risk of CVD events remains unclear. Large weight loss following bariatric surgery is associated with a reduced risk of mortality and CVD among people with type 2 diabetes [3]. However, most individuals with type 2 diabetes do not receive bariatric surgery but are typically given weight management advice including education on healthy behaviour change. Such advice may result in weight maintenance or other changes in weight. However, the long-term effects of advice and changes in weight on CVD and mortality remain uncertain. Glucose-lowering medications that cause weight loss have shown cardiovascular benefits in some instances; however, these benefits may occur through other pleiotropic pathways [4, 5]. As weight management advice and support are integral to diabetes prevention and treatment, the impact on long-term health should be considered. In order to synthesise the existing evidence, we performed a systematic review of research on the association between weight changes and risk of CVD events and mortality among adults with type 2 diabetes. We aimed to summarise evidence regarding the impact of weight loss achieved through behaviour modification on the incidence of CVD and premature mortality, which may inform advice to practitioners and patients about weight management after diabetes diagnosis.

## Methods

### Protocol registration

The protocol was registered on PROSPERO (CRD42019127304) prior to article screening, based on the PRISMA statement [6].

### Study eligibility

Studies eligible for inclusion recruited adults (aged ≥18 years) with a diagnosis of type 2 diabetes. We included observational studies that reported associations between change in weight/BMI (or other metrics of body composition) and CVD events and/or all-cause mortality. Weight data had to be collected at least twice to ascertain changes. Studies reported on CVD events (non-fatal CVD events, CVD mortality, myocardial infarction [MI], stroke, congestive heart failure [CHF]) and/or all-cause or CVD-related mortality. No exclusion criteria were applied related to study design. We did not include studies of weight changes following bariatric surgery as bariatric surgery may affect CVD risk through mechanisms other than weight loss (e.g. alterations to the gut microbiome [7–9]). Similarly, trials of medications that influence weight were excluded as such medications may have pleiotropic effects on CVD risk. Studies including participants without diabetes were included if the results were presented separately for the participants with diabetes.

We included trials of the effects of behavioural interventions on CVD events among adults with type 2 diabetes in which the primary outcome included CVD events and/or mortality. The intervention needed to target physical activity and/or diet for the purpose of weight loss. No restrictions were placed on intervention duration, delivery mode or trial design. Eligible comparisons were as follows: (1) no intervention/minimal intervention; (2) standard care; and (3) other behavioural programmes.

Studies needed to report cardiovascular events including MI, stroke and other CVD-related death. Incident CVD events could occur any time after the intervention period or after the time period in which weight change was assessed. Only published peer-reviewed research articles in the English language were included.

### Search strategy and selection criteria

Searches of the electronic databases MEDLINE, EMBASE, Web of Science, CINAHL and The Cochrane Library (CENTRAL) from inception to 9 July 2019 were conducted by EL and JS with input from a medical librarian. Searches included the following keywords and subject headings: (1) diabetes AND; (2) CVD events AND; (3) weight AND; and (4) study design. Searches were based on the MEDLINE search strategy (electronic supplementary material [ESM] Table 1), modified to include database-specific terms. Terms for ‘study design’ were based on the SIGN search filters [10]. We searched reference lists of eligible studies and review articles to identify additional articles.

We imported search results into Covidence review management software [11]. Two reviewers (of JS, EL, YW, AE and GN) independently screened all abstracts. Full texts were screened for articles deemed to have potential eligibility or where eligibility was unclear. A third reviewer adjudicated if there was uncertainty or disagreement. For both screening stages, all researchers independently piloted an identical 10% of articles.

### Data extraction

Data on participant characteristics, weight assessment, interventions, analysis methods and results were independently extracted by JS and a second reviewer (EL, YW, AE or GN) using a template adapted from the Cochrane data extraction form [12], the Consolidated Standards of Reporting Trials 2010 statement [13] and the Template for Intervention Description and Replication checklist [14]. If details of the methods or study design were not available in the articles, we extracted information from related publications from the same study.

### Risk of bias assessment

Two researchers (JS, EL, YW, AE or GN) assessed studies independently using the Risk of Bias 2.0 tool or a modified version of the Risk of Bias in Non-randomised Studies of Interventions (ROBINS-I) tool, depending on study design. We adapted the ROBINS-I tool to suit observational studies and considered bias related to the following factors: confounding; selection of participants; weight assessment; diabetes assessment; missing data; measurement of outcomes; and study design. Based on these criteria, studies were determined as being at ‘low’, ‘moderate’, ‘serious’ or ‘critical’ risk of bias, or were designated as ‘no information’. The tools were piloted by all researchers for three studies (15%) to ensure consistency. Results are presented using robvis [15].

### Synthesis of results

We meta-analysed the HRs from the intervention studies using a DerSimonian and Laird [16] random effects model and summarised heterogeneity between the studies using the *I*^2^ statistic. We did not meta-analyse the observational studies due to the heterogeneity of study designs and outcomes and instead conducted a narrative synthesis. Forest plots were generated using Stata (Version 16.1; StataCorp, College Station, TX, USA).

## Results

**Fig. 1:**
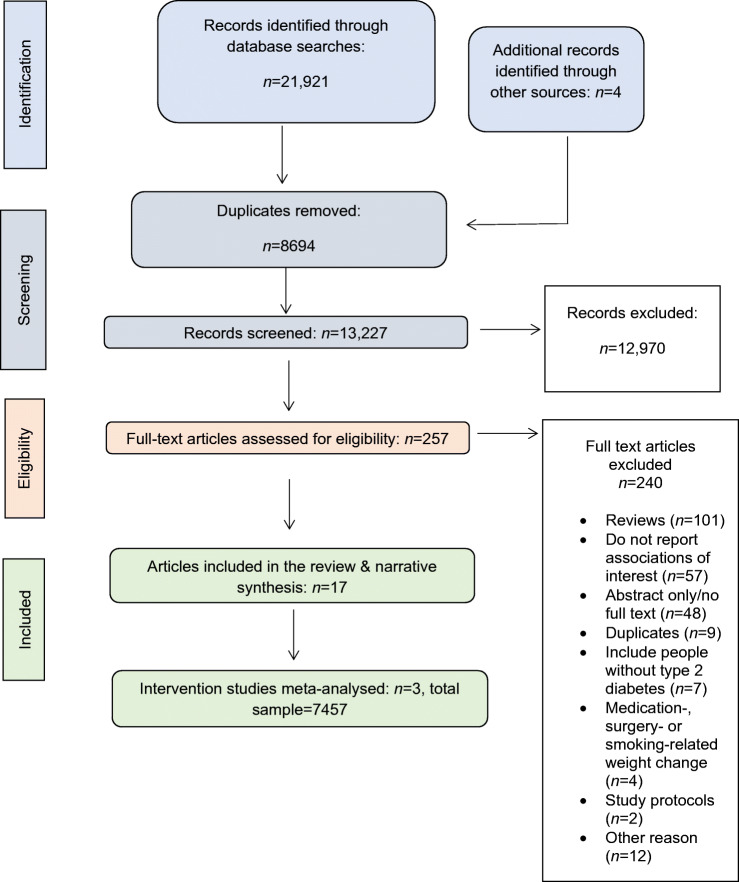
PRISMA diagram

We screened 13,227 titles and abstracts and assessed 257 full text articles. Four additional articles [17–20] were identified from review reference lists. In total, 17 articles [17–33] met our inclusion criteria (Fig. 1). The main reasons for the exclusion of articles were that they did not contain original research or did not report on CVD events or mortality.

### Study characteristics

Fourteen [17–30] of the studies were observational studies, and three [31–33] were RCTs. One study [22] consisted of a post hoc analysis pooling primary data from three clinical trials [34–36]. Studies were conducted in Europe [21–23, 25, 28, 30, 31], the USA [17–20, 22, 24, 26, 29, 33], Japan [32] and South Korea [27], and one study was multinational [22] (Table 1).

**Table 1:** Characteristics of included studies

Study	Study name or cohort	*N*	Diabetes duration	Duration of weight change	Duration of follow-up	Definition of weight change (or intervention and comparators)	Outcomes
Observational studies							
Aucott et al, 2016 [21]	SCI-DC	20,856	0 (new diagnosis)	Diagnosis to 5 years after	Median 5.2 years	Stable-steady, stable-cyclic, gain, loss (referent)	All-cause mortality, CVD events
Bangalore et al, 2018 [22]	CARDS/ASPEN/TNT	6408	Mean~ 8 years	Weight measured 3, 6 and 12 months after baseline and every 6 months thereafter Median of 12 (range 2–15) measurements per participant at varying intervals	Median ranged from 3.9 to 4.9 years	Quintiles of body weight variability (average absolute difference between successive weight values)	Any coronary event, any CVD event, all-cause mortality, CVD mortality
Bodegard et al, 2013 [23]	ROSE	8486	0	18 months	Median 4.6 years	BMI increased by ≥1 kg/m^2^, BMI remained unchanged (+1 to −1 kg/m^2^) (referent), BMI decreased by ≥1 kg/m^2^	CVD-related mortality
Cho et al, 2002 [24]	Nurses Health Study	5897	7–13 years	Difference between weight before diagnosis of diabetes and current weight (updated biennially)	Mean 9.8 years	Gained ≥2 kg, weight change ≤2 kg (referent), lost 2–10.9 kg, lost ≥11 kg	CVD events
Doehner et al, 2012 [25]	PROactive	5202	Median 8 years	2 years	Mean 2.9 years	1% overall weight gain and weight loss, and 1% weight gain and weight loss in the first year in study	All-cause mortality, CVD mortality
Gregg et al, 2004 [19]	NHIS	1401	Mean 10.5 years	1 year	Total 9 years	Lost ≥9.1 kg; lost 0.5–9.0 kg; weight change <0.5 kg (referent); gained 0.5–9.0 kg; gained ≥9.1 kg^a^	All-cause mortality
Gregg et al, 2016 [26]	Look AHEAD	4834	Mean 7 years	1 year	Mean 10.2 years	<2% weight lost or gained (referent), lost >2% to <5%, lost >5% to <10%, lost >10%	CVD events
Hanson et al, 1996 [20]	Gila River Indian Community	572	Not provided	2+ years	Median 8.3 years	High weight fluctuation vs low weight fluctuation (average individual weight fluctuation, accounting for the linear trend in weight fluctuation over time)	CVD events, all-cause mortality
Kim et al, 2019 [27]	Korean National Health Insurance System	173,246	0	2 years	Median 9.3 years	Lost ≥10%, lost ≥5% to <10%, weight change <5% (referent), gained ≥5% to <10%, gained ≥10%	CVD events or all-cause mortality
Koster-Rasmussen et al, 2016 [28]	DCGP	444	0	6 years	Total 13 years	Average annual weight change (kg/year) Categories: aberrant weight pattern; intention to lose weight; intention to maintain weight; intention for weight change not well-described	All-cause mortality, cardiovascular mortality, cardiovascular morbidity
Nunes et al, 2017 [29]	Optum EHR database	143,635	Not provided	1 year	1 January 2009 through 31 December 2014	Lost ≥5%, lost 2–5%, lost 0.5–2%, weight change ≤0.5%, gained 0.5–2%, gained 2–5%, gained 5–7%, gained >7%	MI, stroke, CHF
Strelitz et al, 2019 [30]	ADDITION-Cambridge	725	0	1 year	Mean 9.8 years	Lost ≥5%; lost 2–5%; <2% change in weight (referent); gained >2%	CVD events, all-cause mortality
Williamson et al, 2000 [17]	ACS-CPS	4970	Not provided	1–9 years	Median 12.9 years for survivors and 7.1 for decedents	No change (referent), unintentional loss, unintentional gain, intentional gain, intentional loss, unknown	CVD or diabetes-related mortality, all-cause mortality
Yeboah et al, 2019 [18]	ACCORD	10,251	Mean 10.8 years	Up to 7 years (mean 3.7 years)	Mean 3.5 years	Continuous body weight variability (average absolute difference between successive values of variability)	CVD, CHF, all-cause mortality
Behavioural intervention trials							
Hanefeld et al, 1991 [31]	Diabetes Intervention Study	1008	0	5 year intervention Instruction to follow a low-energy diet and two group teaching sessions on physical activity Annual group sessions promoting activity	Total 5 years	Regular clinical check-ups with 3- to 4-monthly visits Prescribed a diet following the recommendations by the Medical Council for Diabetes Research	CVD and all-cause mortality
Sone et al, 2010 [32]	Japan Diabetes Complications Study	1304	Mean 10.9 years	7 year intervention Face-to-face counselling on diet and activity levels, telephone follow-up every 2 weeks, self-monitoring via diary and pedometer	Median 7.8 years	Regular specialists’ care Continued previous treatment Dietary advice by an administrative dietitian	CVD
Wing et al, 2013 [33]	Look AHEAD	5145	Median 5 years	4 year intervention First 6 months: three group sessions and monthly individual sessions Next 6 months: monthly group and individual sessions In-person and telephone contact monthly onwards	Median 9.6 years	Diabetes support and education	CVD

^a^Weight change category values reported in kg but measured in lb: lost ≥20 lb; lost 1–19 lb; gained 1–19 lb; gained ≥20 lb; referent: weight change <0.5 kg (<1 lb)

ACS-CPS, American Cancer Society Cancer Prevention Study; ADDITION, Anglo-Danish-Dutch Study of Intensive Treatment In People with Screen Detected Diabetes in Primary Care; ASPEN, Atorvastatin Study for Prevention of Coronary Heart Disease Endpoints in non-insulin-dependent diabetes mellitus; CARDS, Collaborative Atorvastatin Diabetes Study; DCGP, Diabetes Care in General Practice; EHR, electronic health records; Look AHEAD, Action for Health in Diabetes; NHIS, National Health Interview Survey; SCI-DC, Scottish Care Information Diabetes Collaboration; PROactive, PROspective pioglitAzone Clinical Trial In macroVascular Events; ROSE, Retrospective Epidemiological Study to Investigate Outcome and Mortality with Glucose-lowering Drug Treatment in Primary Care; TNT, Treating to New Targets

### Participant characteristics

Sample sizes ranged from 444 [28] to 173,246 [27]. Baseline mean age ranged from 46 to 64 years. Diabetes duration was ≤1 year in five studies [21, 23, 27, 28, 30], >1 year in six studies [18, 19, 22, 24–26], and was not specified in three studies [17, 20, 29]. In most studies, diabetes status was derived from medical records, aside from two studies [20, 30] that included screening for type 2 diabetes at enrolment and three studies [17, 19, 24] that relied on participant report of diabetes diagnosis.

Seven studies [22–24, 27, 28, 31, 32] excluded individuals with a history of CVD, whereas inclusion criteria in two studies specified high CVD risk [18, 25]. Other studies excluded individuals with BMI <25 kg/m^2^ [21, 26, 33], history of type 1 diabetes [25, 29] and history of cancer diagnosis [21, 24, 28, 31] (ESM Table 2). One study included women only [24].

### Characteristics of behavioural interventions targeting weight loss

All three RCTs [31–33] evaluated behavioural interventions with diet and physical activity components. One three-armed study [31] also included a pharmacological component; however, one treatment group only received the behavioural intervention, which we compared with the control arm. The intervention durations ranged from 4 years [33] to 8 years [32], delivered via face-to-face individual- and group-based sessions and one also included telephone counselling [32]. The comparator groups received conventional diabetes treatment [31, 32], and the comparator in one study also received education-based weight-loss support delivered in group sessions [33]. Hanefeld et al [31] and Sone et al [32] reported no changes in weight following the intervention. Wing et al [33] reported greater weight loss in the intervention vs control group (8.6% vs 0.7% at 1 year).

### Ascertainment and classification of weight changes in observational studies

Weight measurements were ascertained from medical records in five observational studies [21, 23, 27–29]. Five studies included objective weight measurement [18, 20, 25, 26, 30], and in three studies participants reported their weight [17, 19, 24]. One study provided no information on how weight was ascertained [22]. Most of the observational studies assessed weight change over a period of 1–2 years [19, 21, 23, 25–30]. For studies in which weight change was assessed over ≥2 years, the time between weight measurements varied from 2 to 9 years [17, 24] and was not stated in one study [24]. In four studies, the duration of weight change varied [17, 18, 20, 22] (Table 1).

Six studies reported absolute weight change (kg or lb) [17, 18, 22, 24, 28, 29], one study reported change in BMI units (kg/m^2^) [23], five studies reported percentage weight change [25–27, 29, 30], and four studies focused on weight variability [18, 20–22]. Most studies (*n* = 9) categorised changes in weight to distinguish weight gain, weight maintenance and weight loss, although definitions of the categories varied [18, 19, 23–27, 29, 30]. Three studies [17, 19, 28] assessed participants’ self-reported intention to lose weight.

### Assessment of cardiovascular events and mortality

Most studies (*n* = 16) reported CVD outcomes, and many (*n* = 11) also reported all-cause mortality. In most studies, events were ascertained from medical records and/or mortality registries [17–24, 26–33]. One study did not provide details on the methods of outcome ascertainment [25]. Several studies [19, 21, 26–28, 30] included lags of 1–2 years between the assessment of weight changes and the risk period for mortality outcomes. The median or mean duration of follow-up for outcome events ranged from 2.9 to 10 years.

### Risk of bias

For the observational studies, risk of bias was rated to be moderate for all studies apart from three that were rated high risk of bias [17, 19, 24]. The main sources of bias were uncontrolled confounding, method of weight assessment, and method of diabetes classification (ESM Fig. 1). Of the three intervention studies, one was rated to have low risk of bias [32] and two moderate risk of bias [31, 33].

### Associations between weight change, CVD and mortality in observational studies

**Fig. 2:**
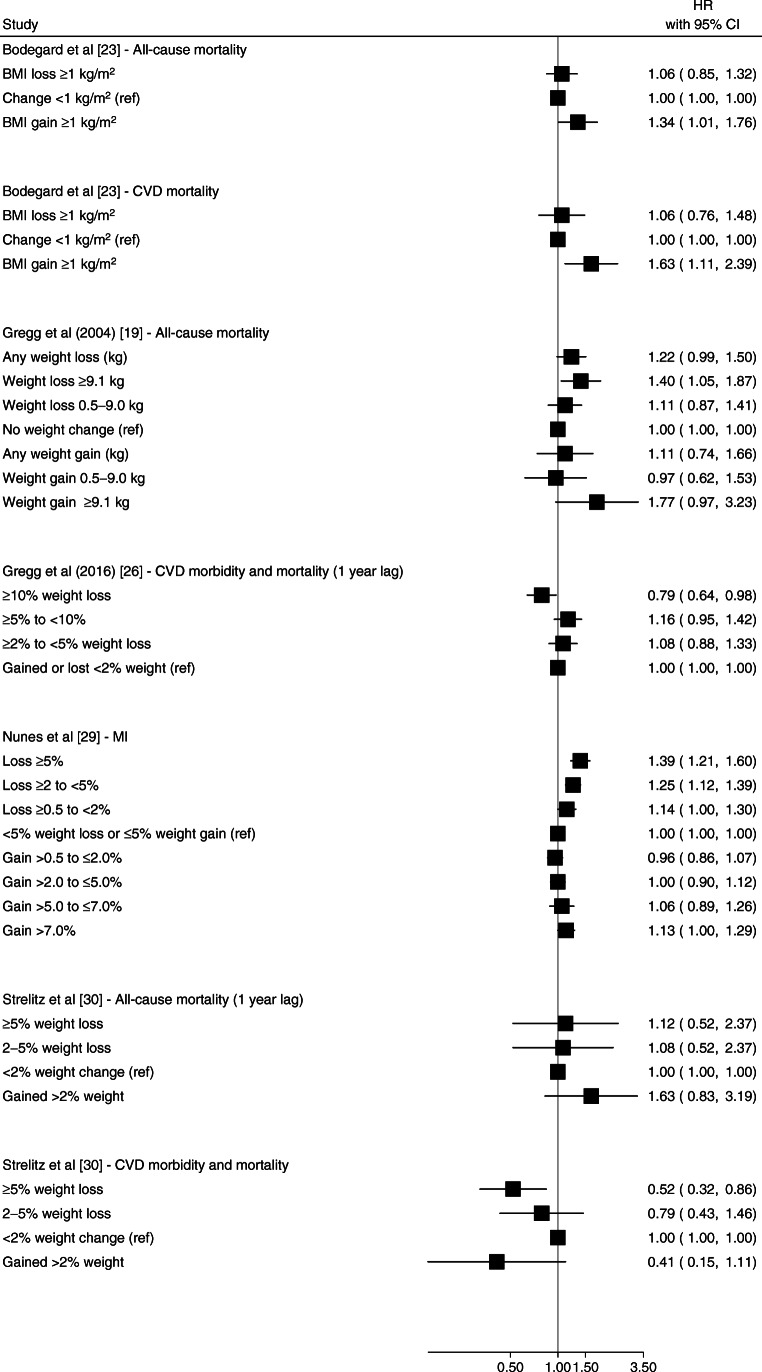
Forest plot of HRs and 95% CIs for the indicated outcome from studies of weight change over 1–2 years. Gregg et al [19] weight change category values reported in kg but measured in lb: lost ≥20 lb; lost 1–19 lb; gained 1–19 lb; gained ≥20 lb; referent: weight change <0.5 kg (<1 lb). Ref, referent group

**Fig. 3:**
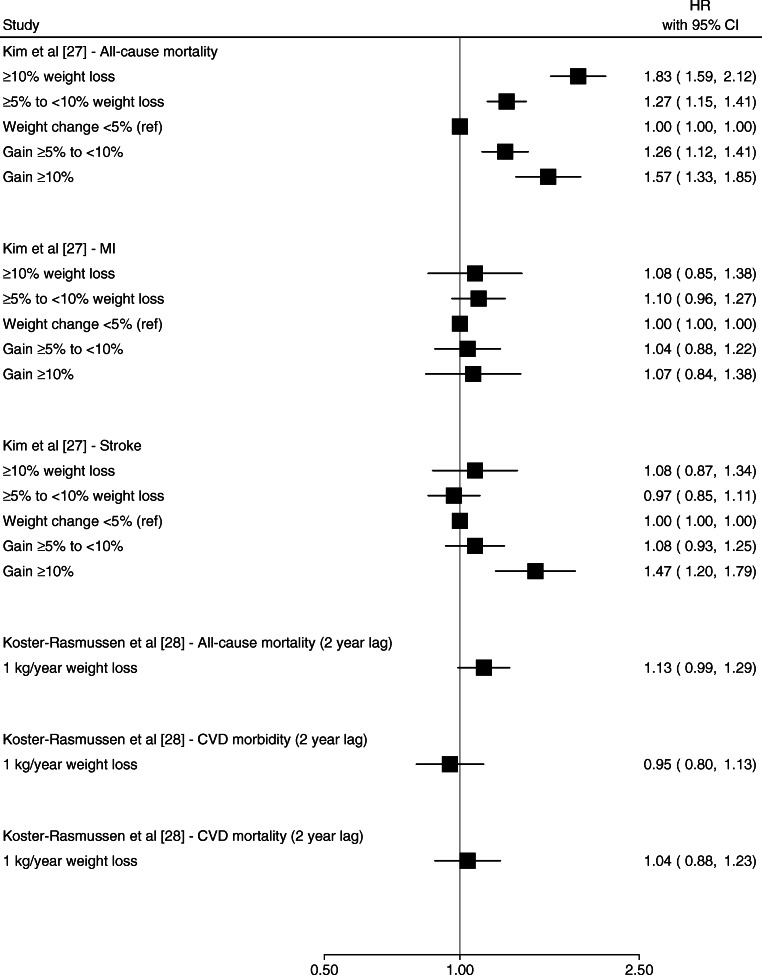
Forest plot of HRs and 95% CIs for the indicated outcome from studies in which the weight change interval was ≥2 years. Ref, referent group

Weight gain was associated with an increased risk of CVD events [23, 27, 29]. Bodegard et al [23] reported that ≥1 kg/m^2^ BMI gain after diabetes diagnosis was associated with higher CVD mortality compared with no BMI change (HR 1.63 [95% CI 1.11, 2.39]) (Fig. 2). Kim et al [27] reported that a ≥10% weight gain after diabetes diagnosis was associated with a higher hazard of stroke (HR 1.47 [95% CI 1.20, 1.79]) and all-cause mortality (HR 1.57 [95% CI 1.33, 1.85]) but not MI events (HR 1.07 [95% CI 0.84, 1.38]) (Fig. 3) [27]. Nunes et al [29] reported that weight gain >7% was associated with higher hazard of MI (HR 1.13 [95% CI 1.00, 1.29]) (Fig. 2) and reported similar results for stroke and CHF [29]. Strelitz et al [30] reported no association between weight gain and CVD events [30] (Fig. 2). Gregg et al [19] reported a higher hazard of all-cause mortality associated with weight gain ≥ 9.1 kg (≥ 20 lb), though the CI overlapped the null (HR 1.77 [95% CI 0.97, 3.23]) (Fig. 2). The duration of follow-up in these studies ranged from 4.6 years to 9.8 years.

Two studies of weight change over <2 years reported protective associations between weight loss and CVD events. Gregg et al [26] and Strelitz et al [30] reported that ≥10% weight loss (vs weight gain or <2% weight loss) and ≥5% weight loss (vs weight change <2.5%), respectively, were associated with a lower hazard of CVD over a mean follow-up of 10.2 years and 9.8 years. In contrast, Nunes et al [29] reported a higher hazard of MI following weight loss ≥5% (vs <5% change) over 5 years, and Gregg et al [19] reported higher 9 year hazard of all-cause mortality for weight loss ≥20 lb (≥9.1 kg) vs no change. Bodegard et al [23] reported no association between a ≥1 kg/m^2^ decrease in BMI over 18 months and risk of CVD mortality over a median of 4.6 years of follow-up (Fig. 2).

**Fig. 4:**
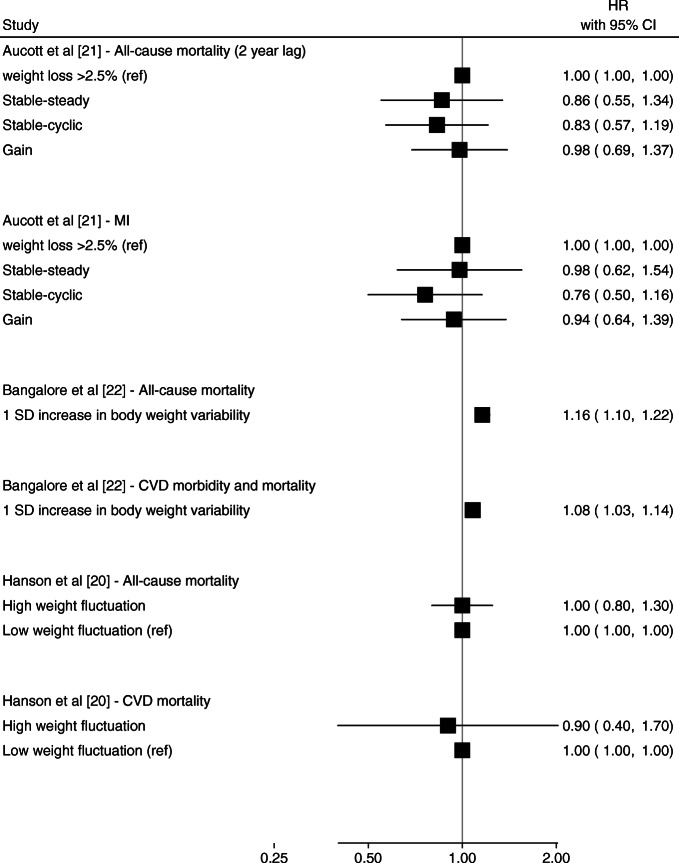
Forest plot of HRs and 95% CIs for the indicated outcome from studies of weight variability over ≥2 years. Ref, referent group

**Fig. 5:**
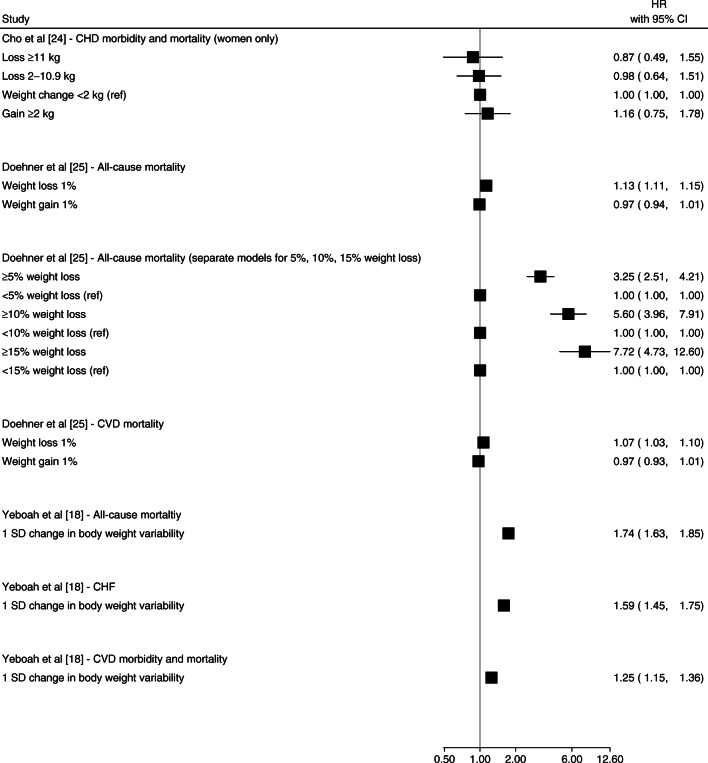
Forest plot of HRs and 95% CIs for the indicated outcome from studies of weight change among individuals with high CVD risk or other subgroups. Ref, referent group

Studies of weight change over 2+ years (Figs. 3 and 4) showed that weight loss and weight variability were associated with CVD events [22] and all-cause mortality [22, 27], particularly among individuals with history of CVD [18, 25] (Fig. 5). Bangalore et al [22] reported small but apparent associations between a 1 SD increase in body weight variability and CVD morbidity and mortality (HR 1.08 [95% CI 1.03, 1.14]) and all-cause mortality (HR 1.16 [95% CI 1.10, 1.22]) over a median 5 years follow-up (Fig. 4) Yeboah et al [18] reported a higher hazard of CHF and CVD per 1 SD difference in body weight variability (HR 1.59 [95% CI 1.45, 1.75] and 1.25 [95% CI 1.15, 1.36], respectively) over a mean 3.5 years of follow-up among individual at high risk of CVD (Fig. 5). Other studies reported null associations between weight loss or weight variability over ≥2 years and CVD events and all-cause mortality [20, 21, 27, 28].

Six studies [19, 21, 26–28, 30] included a 1–2 year lag in the risk period for all-cause mortality as a method for potentially attenuating the confounding effects of unintentional weight loss. In general, studies that included a lag showed null associations between weight change and mortality [19, 26, 28, 30], aside from one study by Kim et al [27] that reported both weight gain and loss (vs weight maintenance) were associated with higher all-cause mortality, with an apparent dose–response relationship between weight change (both gain and loss) and mortality (Fig. 3).

### Intentional and unintentional weight change

**Fig. 6:**
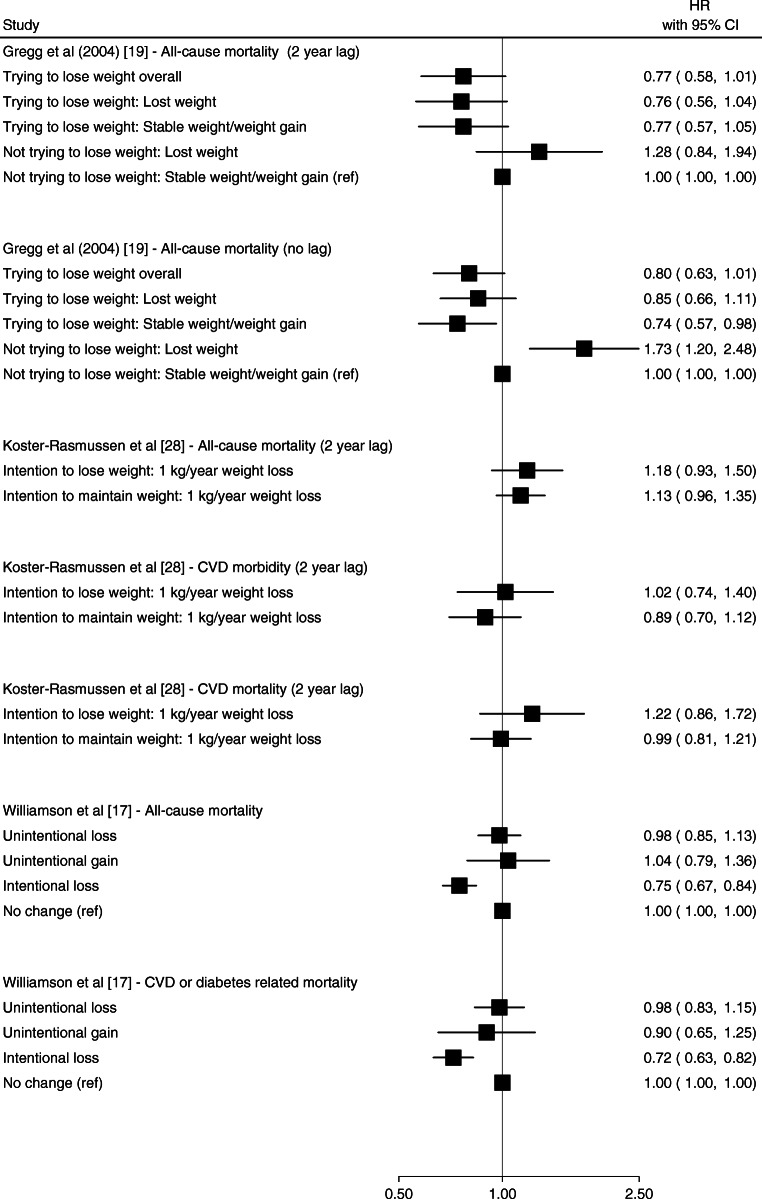
Forest plot of HRs and 95% CIs for the indicated outcome from studies of intentional vs unintentional weight change. Ref, referent group

Three studies assessed the impact of intentional vs unintentional weight changes on CVD and mortality outcomes by categorising participants based on their intention to lose weight [17, 19], or intention to lose or maintain weight [28]. Gregg et al [19] reported that weight loss among participants without intention to lose weight was associated with a higher hazard of all-cause mortality (HR 1.73 [95% CI 1.20, 2.48]), though this association was attenuated after introducing a 2-year lag in the risk period for the outcome (Fig. 6). In the same study, participants who reported that they were trying to lose weight, but did not actually lose weight, had a lower hazard of all-cause mortality compared with participants who were not trying to lose weight (HR 0.74 [95% CI 0.57, 0.98]). This study was determined to have high risk of bias related to confounding and self-reported assessment of weight and diabetes status (ESM Fig. 1). Koster-Rasmussen et al [28] reported no differences in the association between intentional vs unintentional weight loss and all-cause mortality, CVD morbidity or CVD mortality (Fig. 6).

Gregg et al [19] reported no association between intentional weight loss and all-cause mortality (HR 0.85 [95% CI 0.66, 1.11]). Williamson et al [17] reported that intentional weight loss was associated with a lower hazard of death due to CVD or diabetes (HR 0.72 [95% CI 0.63, 0.82]) and all-cause mortality (HR 0.75 [95% CI 0.67, 0.84]). Both studies were determined to have a high risk of bias related to confounding, assessment of weight, and missing data (ESM Fig. 1).

### Population subgroups: high CVD risk and women only

In a study among participants with cardiovascular comorbidities, Doehner et al [25] reported a small increased risk of CVD mortality associated with 1% weight loss over 2 years (HR 1.07 [95% CI 1.03, 1.10]) and increasingly stronger relationships between weight loss ≥5%, ≥10% and ≥ 15% and all-cause mortality (Fig. 5). Yeboah et al. reported that among the Action to Control Cardiovascular Risk in Diabetes (ACCORD) study cohort of individuals with type 2 diabetes and high CVD risk, higher 7-year body weight variability was associated with higher hazard of CVD morbidity and mortality over a mean 3.5 years follow-up [18]. Among women enrolled in the Nurse’s Health Study, Cho et al [24] reported no associations between weight gain or loss, where the duration of weight change was undefined, and risk of CVD over approximately 10 years of follow-up (Fig. 5).

### Effect of interventions targeting weight loss on CVD and mortality

**Fig. 7:**
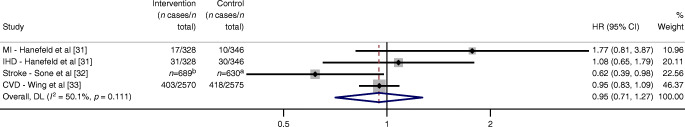
Forest plot of meta-analysis of HRs and 95% CIs from trials of behavioural interventions and incidence of CVD events, by study and outcome. IHD, ischaemic heart disease. Weight (%) shows the relative percentage contribution of each study result to the meta-analysis. Vertical dashed line indicates the meta-analysis point estimate. DL, DerSimonian and Laird method [16]. ^a^Control group event rate: 9.52 events per 1000 person-years. ^b^Intervention group event rate: 5.48 events per 1000 person-years

Meta-analysis showed that behavioural interventions targeting weight loss had no effect on CVD outcomes (HR 0.95 [95% CI 0.71, 1.27]), and heterogeneity was moderately high between studies (*I*^2^ = 50.1%) (Fig. 7). Of the three studies identified [31–33], one behavioural intervention reduced hazard of stroke by 38% (HR 0.62 [95% CI 0.39, 0.98]) over a median 7.8 years follow-up [32]. Wing et al found no effect on CVD incidence (HR 0.95 [95% CI 0.83, 1.09]) over a median of 9.6 years follow-up [33]. Hanefeld et al reported that the MI incidence rate was higher in the intervention group (53.6 per 1000) vs the control group (30.3 per 1000) (rate ratio 1.77 [95% CI 0.81, 3.87]) over a total of 5 years, and found no effect of the intervention on ischaemic heart disease risk (1.08 [95% CI 0.65, 1.79]) [31] (Fig. 7). CIs for the rate ratio were manually calculated based on the numbers of events, as SEs were not provided in the publication.

## Discussion

Behavioural interventions targeting weight loss among people with type 2 diabetes had no effect on 5–10 year risk of CVD outcomes. Synthesis of observational studies showed that weight gain after diabetes diagnosis was associated with higher hazards of stroke [27], CVD mortality [23, 28] and all-cause mortality [19, 27]. However, studies showed mixed results regarding the associations between weight loss and incidence of CVD events and all-cause mortality. The only studies showing protective associations between weight loss and CVD were those concerning weight loss occurring over 1 year [26, 30]. Studies of weight loss or weight variability over longer durations showed associations with increased risks of all-cause mortality [18, 19, 22, 27], although such risks were attenuated in some cases with the introduction of a 1–2 year lag in the risk period. Associations with mortality risks were more consistent in populations with elevated CVD risk [18, 25], and where weight loss was unintentional [17, 19]. Differences in the timing and extent of weight changes, the study populations with respect to CVD risk and diabetes progression, the method to mitigate confounding by unintentional weight loss, and the duration of follow-up contributed to heterogeneity in the results.

Weight loss intention influenced the effect of weight changes on health outcomes. However, while unintentional weight loss appeared to be associated with higher all-cause mortality risk, there was little evidence of protective effects of intentional weight loss. Weight loss intention was self-reported [17, 19, 28] and may not capture weight management behaviours, as details on changes in behaviours were not included in the studies. Participant-reported intention to lose weight does not rule out potential confounding by weight loss caused by underlying disease. Furthermore, the high risk of bias identified in two of the studies that focused on weight loss intention [17, 19] limits interpretation. Other studies attempted to address confounding by unintentional weight loss by including a lag in the risk period for mortality. Typically, the lag attenuated the results, suggesting that unintentional weight loss may otherwise inflate positive associations between weight loss and mortality.

Intervention studies are unlikely to be confounded by unintentional weight loss, although results were still inconsistent. One [33] of the three included trials reported significant weight loss among the intervention group, but nevertheless, the study reported no reduction in CVD events. As the interventions were similar, the heterogeneous findings may be related to differences in study populations, the achievement and maintenance of weight loss, and duration of follow-up. While Wing et al [33] reported no overall association between the behavioural intervention and incidence of CVD, post hoc stratified analyses showed that weight loss among the intervention group was associated with a lower hazard of CVD events [26]. Other post hoc research in this cohort identified heterogeneous intervention effects, depending on participants’ glycaemic control and self-rated health [37, 38]. This heterogeneity may have contributed to the lack of an overall association seen in the original study results. Null findings may also be a product of low statistical power, and it is possible that longer duration of follow-up may be needed to identify the effects of interventions on CVD events in populations that are not specifically at high risk for CVD. For example, a reduction in CVD events was observed only after 23 years of follow-up in the Da Qing Diabetes Prevention Outcome Study [39], and in the Diabetes Prevention Program Outcomes Study, the investigators were not able to assess the effect of the intervention on CVD risk after 10 years of follow-up due to an insufficient number of events [40]. So, the 5–10 year follow-up of the included trials may not have been adequate to assess effects of behavioural interventions on CVD. Furthermore, the heterogeneity in the results between the studies should be considered when interpreting the meta-analysis. It is possible that other factors, including diabetes severity or participant characteristics, may influence the effect of weight-loss interventions on CVD and mortality outcomes but this has not been assessed. Risk of bias in the intervention studies was determined to be moderate in two and low in one (ESM Fig. 2), so bias is unlikely to be the primary reason for discrepant results between the trials. As we identified only three eligible studies that reported CVD events, this review highlights the need for more behavioural intervention studies with long-term follow-up.

In the observational studies, associations between weight change, CVD and mortality were influenced by baseline CVD risk in the study population. Two studies using data from high-CVD-risk groups (ACCORD [18] and PROactive [25]) reported associations between weight loss, weight variability and higher risk of mortality. However, the ACCORD trial intervention included intensive use of glucose-lowering medication, and the observed associations may have been confounded by the effects of these medications on weight and CVD risk. While weight loss may have adverse effects among high-risk groups, it is also possible that the larger number of CVD events and higher premature mortality risk in these cohorts facilitated detection of associations between weight loss and events, which would be more difficult to detect in other cohorts. The results suggest that future behavioural intervention trials should consider testing effects of interventions on CVD separately among subgroups at higher CVD risk.

Studies of weight changes occurring over 1–2 years were more likely to show protective associations with CVD, while weight changes occurring over longer periods of time more often reported adverse associations [18, 22, 27]. This may be due to the fact that the majority of the studies with longer weight change duration did not include a lag time in the risk period for outcomes and may have been more susceptible to confounding by unintentional weight loss. Studies did not assess heterogeneity by differences in baseline BMI or by age, as has been done in other studies of the health effects of weight loss in the general population [41, 42]. Type 2 diabetes ascertainment was typically based on clinical diagnosis and did not include biochemical assessment of rare diabetes subtypes. Most studies did not exclude individuals with a history of cancer diagnosis, and it is possible that unintentional weight loss may be more common in this group.

We were unable to perform a meta-analysis of the observational studies due to heterogeneity in multiple study characteristics. Differences in study populations, outcomes, exposures and baseline CVD risk also complicated the narrative synthesis. As we only included published peer-reviewed research, this review is subject to publication bias if relevant unpublished works were excluded. We only included studies that included CVD events or mortality as an outcome; therefore, this review is not inclusive of behavioural intervention trials that focus on intermediate outcomes such as cardiovascular risk factors.

While there is compelling evidence that weight loss following bariatric surgery reduces risk of CVD [3], we have shown that the impact of non-surgical weight loss on CVD and mortality remains unclear. Weight gain was consistently associated with higher risk of CVD events but findings for the impact of weight loss are conflicting. Trial evidence of the effect on CVD endpoints of behavioural interventions targeting weight loss was also inconsistent and it remains unclear which subgroups of patients benefit most.

### Conclusions

Preventing weight gain among people with type 2 diabetes may help to reduce long-term burdens of CVD and premature mortality. While evidence accumulates for the benefits of weight loss on risk factors in the short term, there is limited evidence that existing behavioural approaches to achieving weight loss deliver long-term cardiovascular health benefits. In order to target weight-loss interventions efficiently, research is needed to identify patient groups that will achieve lower CVD event rates following weight loss, and to determine how much weight loss should be achieved and for how long this should be maintained. This evidence will help to most effectively allocate resources to improve long-term outcomes for people with diabetes.

## Supplementary information

ESM 1 (PDF 260 kb)

## Data Availability

The data from this study are available from the corresponding author on reasonable request.

## Abbreviations

ACCORD: Action to Control Cardiovascular Risk in Diabetes
CHF: Congestive heart failure
MI: Myocardial infarction
ROBINS-I: Risk of Bias in Non-randomised Studies of Interventions
