# Supplementary material for: Association between weight change and incidence of cardiovascular disease events and mortality among adults with type 2 diabetes: a systematic review of observational studies and behavioural intervention trials
Source: Diabetologia. 2021 Dec 2;65(3):424–39. doi: 10.1007/s00125-021-05605-1 (PMC8803822; doi:10.1007/s00125-021-05605-1)

ESM Table 1. Medline search terms

1	diabetes mellitus, type 2/
2	(NIDDM or T2D*).tw.
3	(non insulin* depend* or noninsulin* depend* or noninsulin*depend* or non insulin*depend*).tw.
4	((typ* 2 or typ* II or typ* two or typ*2 or typ*II or typ*two) adj2 diabet*).tw.
5	1 or 2 or 3 or 4
6	Body Weight/
7	Body Mass Index/
8	Waist Circumference/
9	Body Weight Changes/
10	Weight Loss/
11	(weight adj2 (body or chang* or loss* or maint* or manag* or control* or reduc* or gain*)).tw.
12	(“body mass index” or bmi).tw.
13	(body adj2 (mass or fat)).tw.
14	(lean adj2 mass).tw.
15	(waist* adj2 (circumferenc* or hip)).tw.
16	6 or 7 or 8 or 9 or 10 or 11 or 12 or 13 or 14 or 15
17	Myocardial Infarction/
18	(Myocardial adj2 infarct*).tw.
19	MI.tw.
20	Cardiovascular diseases/
21	(Cardiovascular adj2 (disease* or event*)).tw.
22	CVD.tw.
23	Heart diseases/
24	(Heart adj2 (disease* or attack)).tw.
25	Coronary disease/
26	(coronary or ischemic adj2 (heart or disease*)).tw.

27	Cerebrovascular Disorders/
28	(cerebrovascular adj2 (disease* or event*)).tw.
29	(Cerebral adj2 infarct*).tw.
30	Stroke.tw.
31	17 or 18 or 19 or 20 or 21 or 22 or 23 or 24 or 25 or 26 or 27 or 28 or 29 or 30
32	5 and 16 and 31
33	Epidemiologic studies/
34	Exp case control studies/
35	Exp cohort studies/
36	Case control.tw.
37	(cohort adj (study or studies)).tw.
38	Cohort analy*.tw.
39	(Follow up adj (study or studies)).tw.
40	(observational adj (study or studies)).tw.
41	Longitudinal.tw.
42	Retrospective.tw.
43	Cross sectional.tw.
44	Cross-sectional studies/
45	Randomised Controlled Trials as Topic/
46	randomised controlled trial/
47	Random Allocation/
48	Double Blind Method/
49	Single Blind Method/
50	clinical trial/
51	clinical trial, phase i.pt
52	clinical trial, phase ii.pt
53	clinical trial, phase iii.pt
54	clinical trial, phase iv.pt

55	controlled clinical trial.pt
56	randomised controlled trial.pt
57	multicenter study.pt
58	clinical trial.pt
59	exp Clinical Trials as topic/
60	(clinical adj trial*).tw.
61	((singl* or doubl* or treb* or tripl*) adj (blind*3 or mask*3)).tw.
62	PLACEBOS/
63	placebo*.tw.
64	randomly allocated.tw.
65	(allocated adj2 random*).tw.
66	(non randomi* or nonrandomi* or non-randomi* adj trial).tw.
67	Or/33-66
68	32 and 67

ESM Table 2. Participant characteristics of included studies

1st author (year)	Country	Study population	Age at baseline, years (mean)	BMI, kg/m2 at baseline	Female; N, %	Recruitment method	Inclusion/exclusion criteria	Ethnicity; N (%)
**Aucott (2016)**	UK (Scotland)	Newly diagnosed T2DM	58.4 (SD=12)	33.2 (SD=6.0)	13486/29316 (46%)	Population-based database derived from primary & secondary care as a register of people with diabetes (Scottish Care Information Diabetes Collaboration (SCI-DC) database)	Inclusion: Adult patients (>18 years); newly diagnosed with T2DM between 2002 & 2006; relevant information for ≥2 years<br><br>Exclusion: Previous diagnosis of cancer, thyroid disease or on oral steroids; BMI <25 kg/m2 at diagnosis; died or moved out of Scotland within the first 2 years after diagnosis; HbA_{1c}; <42 mmol/mol (6.0%) at diagnosis or prescribed insulin within 18 months of diagnosis; only one recorded weight measure or with <21 months follow-up	Primarily White European
**Bangalore (2018)**	Multi-country (UK, Ireland, USA, Australia, Austria, Canada, Finland, France, Germany, Italy, the Netherlands, New Zealand, Norway, South	3 clinical trials of statins	Overall: 61.7	Baseline Overall: 29.2 (±4.1) (85.1(±14.7) kg).<br><br>CARDS 28.8 (±3.6) ASPEN 28.9 (±3.8) TNT 30.4 (±5.3)	2032/6408 (31.7%)	Eligible individuals were identified through medical records. Investigators from the clinical centres review of patient registries. Opportunistic recruitment was conducted among patients attending assessment at diabetes clinics	Inclusion: Type 2 diabetes mellitus at baseline enrolled in 3 clinical trials of statins if they had at least 2 post-baseline measurements of body weight.<br><br>Exclusion: TNT: survival-limiting disease; unexplained creatine phosphokinase levels >6 times the upper limit of normal; concurrent therapy with long-term immunosuppressants; concurrent therapy with lipid-regulating drugs not specified as study treatment in the protocol; history of alcohol abuse; and participation in another clinical trial concurrently or within 30 days before screening. ASPEN: type 1 diabetes; history of CVD; HbA_{1c} >86 mmol/mol (10%); active liver disease or hepatic dysfunction; severe renal dysfunction or nephrotic syndrome;	CARDS: White ethnic origin (n=2676; 94%)<br><br>Not reported for other studies

	Africa, Spain, Switzerland)						congestive heart failure treated with digoxin; creatine phosphokinase ≥3 × the upper limit of normal; blood pressure >160/100 mmHg; BMI >35 kg/m2; abuse of alcohol and/or drugs; hypersensitivity to the study medication; participation in another clinical study within 30 days of screening; placebo run-in compliance rate <80%; current or planned pregnancy; or use of excluded medications. These medications included immunosuppressive agents, drugs known to interact with the study medications or affect clinical laboratory parameters (e.g., systemic steroids or isotretinoin), and drugs associated with increased risk of rhabdomyolysis with statins (e.g., cyclosporine and macrolide antibiotics). Subjects taking lipid-altering medications, including other statins, were screened after a 4-week washout phase. CARDS: plasma creatinine concentration greater than 150 μmol/L, HbA_{1c}>108 mmol/mol (12%), during the baseline phase they had less than 80% compliance with placebo.	
**Bodegard (2013)**	Sweden	Newly diagnosed T2DM & no history of CVD or cancer (all receiving glucose lowering drug treatment in primary care)	Overall: 60.0 (range: 35-79<br>Increased BMI: 57.9 (10.2)<br>Unchanged BMI: 59.7 (10.1)	Overall: 30.2 (range 16.7-58.5)<br>Increased BMI: 30.1 (5.8)<br>Unchanged BMI: 30.2 (5.1)	Overall: n=3816/8486 (45.0)<br>Increased BMI: n=518/1238 (41.8)<br>Unchanged BMI: n=1852/4523 (40.9)	ROSE study (trial of glucose lowering drug treatment in primary care) - Patients' data extracted from 84 primary-care centres in 2010	Inclusion: Patients diagnosed with T2DM between 1999-2009 & no previous history of CVD or cancer.<br>Exclusion: Previous history of prevalent diabetes from 1987 up to the data extraction date; aged either less than 35 years or more than 79 years; newly diagnosed with diabetes after 2008.	Not reported

			Decreased BMI: 59.0 (10.3)	Decreased BMI: 32.7 (5.8)	Decreased BMI: n=1446/2725 (53.1)			
**Cho (2002)**	USA	Women with diabetes aged ≥40	BMI less than 23: 54<br>BMI of 23.0–24.9: 54<br>BMI of 25.0–26.9: 56<br>BMI of 27.0–29.9: 55<br>BMI of 30.0–34.9: 55<br>BMI of 35.0 or more:54<br>At midpoint of follow-up (in 1986, 10 years from baseline)	BMI less than 23: n=633<br>BMI of 23.0–24.9: n=444<br>BMI of 25.0–26.9: n=473<br>BMI of 27.0–29.9: n=689<br>BMI of 30.0–34.9: n=847<br>BMI of 35.0 or more: n=593<br>At midpoint of follow-up (in 1986, 10 years from baseline)	N=5,897 (100%)	Nurse's Health Study - established in 1976; 121,700 female registered nurses, aged 30–55 years in 11 U.S. states returned a self-report questionnaire	Inclusion: information on both height and weight in 1976; Aged 40 - 74 years; physician's diagnosis of diabetes at 40 years of age or older<br>Exclusion: history of cardiovascular disease (including MI, angina, coronary revascularisation, and/or stroke) and/or cancer (except non-melanoma skin cancer) in 1976; For women in whom diabetes was diagnosed after 1976, developed cardiovascular disease or cancer before or at the time of diabetes diagnosis.	96.7% White; 3.3% Other (2% African-Americans, 0.5% Hispanics & 0.8% Asians)
**Doehner (2012)**	19 European countries (Austria, Belgium, Czech Republic, Denmark, Estonia,	T2DM & pre-existing cardiovascular co-morbidity	62 (SD 8) (range: 35-75)<br>Pharmacology: 61.9 (7.6)<br>Placebo: 61.6 (7.7)	Overall 30.9 (4.8) (for PROactive study n=5238)	N=1765/5202 (33.9%)<br>Pharmacology: 867/2592 (33.4)<br>Placebo: 898/2610 (34.4)	PROactive study - patients from primary-care practices & diabetic or cardiovascular specialist	Inclusion: Pre-existing CVD (defined as myocardial infarction or stroke at least 6 months before entry to the trial, percutaneous coronary intervention or coronary artery bypass surgery at least 6 months before recruitment, acute coronary syndrome at least 3 months before recruitment, or objective evidence of coronary artery disease or obstructive arterial disease	White: 5130 (98.6%)

	Finland, France, Germany, Hungary, Italy, Latvia, Lithuania, Netherlands, Norway, Poland, Slovakia, Sweden, Switzerland, UK)			Pharmacology: 30.7 (4.7) (87.5kg (15))<br><br>Placebo: 31.0 (4.8) (88.6kg (16))		departments in hospitals	in the leg); aged 35-75; HbA_{1c} >48mmol/mol (6.5%) despite anti-diabetic therapy<br><br>Excluded: type 1 diabetes; taking only insulin; planned coronary or peripheral revascularisation; had New York Heart Association class II heart failure or ischemic ulcers or gangrene, leg pain at rest, or were on haemodialysis or had greater than 2·5 times the upper limit of normal concentrations of alanine aminotransferase	
**Gregg (2004)**	USA	Physician-diagnosed diabetes 1989-1997.	total mean 61.2, None: 62.1, loss: 60.1, gain:62.4, Not trying:64.2, yes trying:59.9	total:31.6 none: 30.5 loss: 33.0 gain: 30.4 no: 29.6 yes:32.6	Total: 57.6%<br><br>None:52.5% Loss:60.9%<br><br>Gain: 66.2%<br><br>No: 44.6%<br><br>Yes: 63.4%	NHIS annual nationwide survey of ~45,000 households & 120,000 individuals (response rate 95%). Used data from 1989.	Inclusion: age ≥18 years, reported physician-diagnosed diabetes, was asked about weight loss and other behaviours and services related to diabetes.<br><br>Exclusion: BMI before weight loss <25 kg/m2, aged <35 years	% Non-White: 22.7%
**Gregg (2016)**	USA	Type 2 diabetes, BMI ≥25 kg/m² (or ≥27 kg/m² for those receiving insulin therapy), ability to complete exercise test.	Range: 45–76 years.<br><br>Gain/stable: 58·4 (6·9); Small loss: 58·9 (6·8); Medium loss: 58·7 (6·8);	Gain/stable: 35·9 (5·8); Small loss: 35·8 (6·0); Medium loss: 35·8 (6·1); Large loss: 36·1 (5·8)	Gain or stable: n=1173 (59%); Small loss: n=556 (61%); Medium loss: n=613 (61%); Large loss: n=568 (56%)	Cohort from multicentre trial with 16 clinical sites in the USA. Recruitment from informational mailings, open screenings,	Inclusion: BMI of at least 25 kg/m² or of at least 27 kg/m² for those receiving insulin therapy.<br><br>Exclusion: HbA_{1c} >97 mmol/mol (11%), systolic blood pressure greater than 160 mm Hg, diastolic blood pressure greater than 100 mm Hg, or plasma triglyceride concentration greater than 600 mg/dL (6·78 mmol/L), or unable to complete a maximal	Gain or stable: Non-White: n=763 (39%), White: n=1209 (61%);<br><br>Small loss: Non-White: n=383 (42%), White: n= 531 (58%);

			Large loss: 59·3 (6·9)			advertisements, and referrals from health care professionals between 08/2001 - 04/2004	graded exercise test or 2 weeks of self-monitoring of diet and activity	Medium loss: Non-White: n= 374 (37%), White: n=626 (63%);<br>Large loss: Non-White: n= 270 (27%) White: n= 742 (73%)
**Hanefeld (1991)**	Germany	30-55 years with newly diagnosed T2DM controlled by diet	Controls: 46.6 (5.6)<br>Intensified health education (IHE) plus placebo: 46.2 (7.0)<br>IHE plus clofibric acid: 45.8 (8.8)	Controls: 28.9 (5.0)<br>IHE plus placebo: 29.2 (5.8).<br>IHE plus clofibric acid: 29.4 (4.7)	Controls: 172/378 (45.5%)<br>IHE plus placebo: 151/382 (39.5%).<br>IHE plus clofibric acid: 181/379 (47.8)	Sixteen diabetes clinics located in urban and rural areas of Germany based on a centralised registration - reasons for fasting blood glucose measurements were casual investigation 55.4%, clinical symptoms 23%, checkup 12.8%, and others 8.8%	Inclusion: Aged 30-55 year; newly detected elevation of fasting blood glucose of >7.21 mM; diabetes controlled by diet after 6 weeks of a conventional diet; oral consent to take part in the study program.<br>Exclusion: myocardial infarction before entry, stroke, gangrene, cancer, other severe life-limiting illness, clofibric acid intake, diet not satisfactory, and not willing or able to perform the IHE program and/or the diagnostic procedures of the project	Not reported
**Hanson (1996)**	USA	Pima Indians with type two diabetes (non-insulin dependent)	Overall: 52 (12)<br>Low root mean square error (RMSE): 53	Overall: NR<br>Low RMSE: 29.3<br>High RMSE: 30.3	Overall: NR<br>Low RMSE:66.4%<br>High RMSE: 59.1%	Community residents ≥5 years of age invited to participate in a research examination	Inclusion: Aged 20 years or over who were community residents at the 4th examination.<br>Exclusion: NR	Pima Indians: 100%

			High RMSE: 51			every 2 years (1995 onwards).		
**Kim (2019)**	South Korea	New onset T2DM who started taking medication within 1 year of their health check-up	By weight change category:<br><br>≥ -10%: 57.2 (12.1)<br><br>-10% ~ -5%: 56.7 (10.9)<br><br>-5 ~ 5%: 56.0 (10.2)<br><br>5 ~ 10%: 54.9 (10.3)<br><br>≥ 10%: 54.2 (10.8)	By weight change category:<br><br>≥ -10%: 69.6kg (13.1)<br><br>-10% ~ -5%: 69.3 (12.1)<br><br>-5 ~ 5%: 69.5 (11.4)<br><br>5 ~ 10%: 66.7 (11.4)<br><br>≥ 10%: 63.3 (11.1)	By weight change category:<br><br>≥ -10%: n=3557/6897 (51.6)<br><br>-10% ~ -5%: n=10891/25468 (42.8)<br><br>-5 ~ 5%: 79477 males (67.0%), 39214 females<br><br>5 ~ 10% - 11418 males (70.8%), 4709 females<br><br>≥ 10% - 4400 males (72.7%), 1653 females	Korean National Health Insurance System	Inclusion: Aged >=30 years between 2007 and 2012; new-onset T2DM who started taking diabetic medication within 1 year of health check-up; not previously taken any diabetic medication; fasting blood glucose >=126 mg/dL at health examination)<br><br>Exclusion: History of MI or stroke before the index year; malignancy	Korean:100%
**Koster-Rasmussen (2016)**	Denmark	Newly diagnosed T2DM & BMI ≥25	Intention to lose: 58.8 (9.6)<br><br>Intention to maintain: 65.4 (10.7)	Intention to lose weight: 32.6 (4.7) (91.6kg (16.1))<br><br>Intention to maintain: 30.3	Intention to lose: 103/209 (49%) male.<br><br>Intention to maintain: 109/210 (52%) male	Newly diagnosed with diabetes in general practices	Inclusion: ≥40 years old; newly diagnosed with type 2 diabetes between 1989-1992; BMI >=25 at time of diabetes diagnosis; at least 3 valid measurements of weight<br><br>Exclusion: History of CVD prior to diabetes diagnosis, history of cancer, death during the 6 year observation period.	NR

				(4.0) (84.0kg (13.3))					
**Nunes (2017)**	USA	Patients with T2DM treated with sulfonylureas (SU) 2009-2014.	<30: n=1333 (0.9)<br><br>30 – 39: n=5420 (3.8)<br><br>40 – 49: n=15645 (10.9)<br><br>50 – 59: n=32796 (22.8)<br><br>60 – 69: n=39852 (27.7)<br><br>>69: n=48491 (33.8)<br><br>Missing: n=98 (0.1).<br><br>Ranged <30 to >69	Median (IQR)<br><br>Overall: 32.3 (28.1, 37.6)<br><br>At least 1 hypoglycaemia: 31.7 (27.4, 37.2)<br><br>No hypoglycaemia: 32.4 (28.1, 37.6)	Overall: N=69,879 (48.7%)<br><br>At least 1 hypoglycaemia: 5961 (48.9%)<br><br>No hypoglycaemia: 63 918 (48.6%)	Electronic health record data system (reflects customary clinical practice in USA -data from over 195 hospitals, 25,000 physicians and 25 million patients)	Inclusion: Patients with type 2 diabetes; received a prescription for an SU with at least 1 year of preceding (baseline) data; at least 1 outpatient visit with an evaluation and management code during the 12 month-baseline period.<br><br>Exclusion: less than 21 years of age; prior diagnosis of type 1 diabetes (at any time) or gestational diabetes (within the prior 12 months)	Overall: Hispanic: n=9920 (6.9%); Non-Hispanic: n=124,929 (87.0%). Other/unknown: n=8786 (6.1%)<br><br>At least 1 hypoglycaemia: Hispanic: n= 1111 (9.1%); Non-Hispanic: n=10411 (85.4%); Other/unknown: n=664 (5.4%)<br><br>No hypoglycaemia: Hispanic: n=8809 (6.7%); Non-Hispanic: n=114518 (87.1%); Other/unknown: n=8122 (6.2%)

**Sone (2010)**	Japan	Patients with T2DM aged 40–70 years with HbA_{1c} levels ≥6.5%.	Overall: 58.5 (6.9)<br><br>Control: 58.6 (7.0)<br>Intervention: 58.5 (6.9)	Overall:<br><br>Control: 23.0 (2.9)<br>Intervention: 23.1 (3.1)	Overall: n= 946/2033 (46.5%).<br><br>Control: n= 478/1,016 (47.0)<br><br>Intervention: n= 468/1,017 (46.0)	Outpatient clinics in 59 university and general hospitals nationwide that specialised in diabetes care.	Inclusion: Previously diagnosed patients with T2DM aged 40–70 years; HbA_{1c} levels were ≥48mmol/mol (6.5%).<br><br>Exclusion: history of angina pectoris; myocardial infarction; stroke; peripheral arterial disease; familial hypercholesterolaemia; type III hyperlipidaemia; non-diabetic nephropathy; nephrotic syndrome; pre-proliferative & proliferative retinopathy; intra-ocular surgeries; serum creatinine levels >120 μmol/l, & mean values of two spot urine examinations for an albumin excretion rate of <150 mg/g creatinine.	Not reported
**Strelitz (2019)**	UK (England)	Year following T2DM diagnosis	Full cohort: 61.1 (7.1)<br><br>Gained >2% weight: 60.8 (7.1)<br><br>Maintained weight: 60.4 (7.5)<br><br>Lost ≥2% to <5% weight: 61.0 (7.3)<br><br>Lost ≥5% weight: 61.8 (6.4)	Full cohort: 33.4 (5.6) (94.6kg(17.6))<br><br>Gained >2% weight: 32.3 (6.3)<br><br>Maintained weight: 32.8 (5.4)<br><br>Lost ≥2% to <5% weight: 33.4 (5.4)<br><br>Lost ≥5% weight: 34.2 (5.6)	Full cohort: n=279/725 (38.5)<br><br>Gained >2% weight: n=30/79 (38.0%)<br><br>Maintained weight: n=71/222 (32.0%)<br><br>Lost ≥2% to <5%: n=64/183 (35.0%)<br><br>Lost ≥5% weight: n=114/241 (47.3%)	Screened detected diabetes from 49 general practices (GPs) in eastern England (invited to enrol in ADDITION trial)	Inclusion: Aged 40–69; Screened to have T2DM per 199 WHO criteria; identified to be at 'high risk for diabetes' based on their medical records using validated risk score using electronic GP records<br><br>Exclusion: Negative screening for type 2 diabetes	97% White European

**Yeboah (2018)**	USA	T2DM & CVD comorbidity.	Overall: 62.8 (6.6)<br><br>BMI ≤25: 64.0 (7.5)<br><br>BMI 25-30: 63.9 (6.9)<br><br>BMI >30: 62.0 (6.3)	Range: 30.4 kg to 50.6 kg.<br><br>Overall: 32.2 (5.4)<br><br>BMI ≤25: N = 911/10251 (8.9%), Mean: 23.5 (1.3)<br><br>BMI 25-30: N = 2985/10251 (29.1%), Mean: 27.7 (1.4)<br><br>BMI >30: N = 6355/10251 (62.0%), Mean: 35.5 (3.9)	Overall: 38.5%<br><br>BMI ≤25: 35.7%; BMI 25-30: 31.6%; BMI >30: 42.2%	Randomised controlled trial that compared intensive blood pressure, glycaemic & lipid treatment with standard care in patients with T2DM	Inclusion: Type 2 diabetes mellitus; HbA_{1C} ≥7.5%; aged 40 to 79 with coronary artery disease or 55 to 79 years with: anatomical evidence of significant atherosclerosis, albuminuria, left ventricular hypertrophy or ≥2 cardiovascular risk factors (dyslipidemia, hypertension, current smoking, and obesity); at least 2 recorded weights<br><br>Exclusion (of ACCORD trial): frequent or recent serious hypoglycaemic events; unwillingness to perform home glucose monitoring or insulin injections; BMI > 45; serum creatinine > 1.5 mg/dL; serious illness.	Overall: White: 62.4%; Non-white: 37.6%<br><br>BMI ≤25: White: 41.8%; Non-white: 58.2%<br><br>BMI 25-30: White: 57.6%; Non-white: 42.4%<br><br>BMI >30: White: 67.5%; Non-white: 32.5%
**Williamson (2000)**	USA	Previous diagnosis of diabetes.	55	Unintentional gain group: 29.9<br><br>Unintentional loss group: 31.8<br><br>Intentional loss group: 33.5	Overall: n=2461/4970 (49.5%)	By the American Cancer Society in 25 states for the baseline interview of the Cancer Prevention Study I	Inclusion: adults 40-64 years old who had a self-reported previous diagnosis of diabetes.<br><br>Exclusion: age > 65 years; age<40 years of age because this age-group had very few deaths, initial BMI <27 kg/m^2.	% Non-White, by weight change category: No change, 4.6%. Unknown, 6.1%. Unintentional gain, 6.4%. Unintentional loss, 5.2%. Intentional loss, 2.6%.

**Wing (2013)**	USA	Overweight or obese patients with T2DM	Overall: 58.7<br><br>Control: 58.9 (6.9)<br><br>Intervention: 58.6 (6.8)	Overall:<br><br>Control: 36.0 (5.8)<br><br>Intervention: 35.9 (6.0)	Overall: n=3087/5145 (60%)<br><br>Control: n=1537/2575 (59.7)<br><br>Intervention: n=1526/2570 (59.4)	Multicentre trial with recruitment from informational mailings, open screenings, advertisements, and referrals from health care professionals between 08/2001 - 04/2004	Inclusion: Aged 45-75; Type 2 Diabetic; BMI of 25+ (27.0 or greater in patients taking insulin); HbA_{1c} <11%; Blood pressure < 160/100; Triglyceride level < 600mg/dl; Ability to complete valid maximal exercise test; established relationship with a primary care provider (% of trial patients using insulin was kept to <30% of the cohort)<br><br>Exclusion: HbA_{1c} ≥97mmol/mol (11%), blood pressure ≥ 160/100 mmHg, triglycerides ≥ 600 mg/dl, inadequate control of pre-existing conditions, factors affecting adherence to the intervention or may conduct of the trial, and underlying disease which may affect safety of the intervention	Black: n=804 (15.6%)<br><br>Native American: n=258 (5%)<br><br>Asian or Pacific Islander: n=50 (0.97%)<br><br>White: n=3252 (63.2%)<br><br>Hispanic: n=680 (13.2%)<br><br>Other: n=101 (1.96%)

ACCORD, Action to Control Cardiovascular Risk in Diabetes; ADDITION, Anglo-Danish-Dutch Study of Intensive Treatment In People with Screen Detected Diabetes in Primary Care; ASPEN, Atorvastatin Study for Prevention of Coronary Heart Disease Endpoints in non-insulin-dependent diabetes mellitus; BMI, body mass index; CARDS, Collaborative Atorvastatin Diabetes Study; CVD ,cardiovascular disease; TNT, Treating New Targets; IQR, inter-quartile ratio; MI, myocardial infarction; T2DM: type 2 diabetes mellitus; HbA_{1c}, glycated haemoglobin

ESM Figure 1. Risk of bias visualisation for observational studies

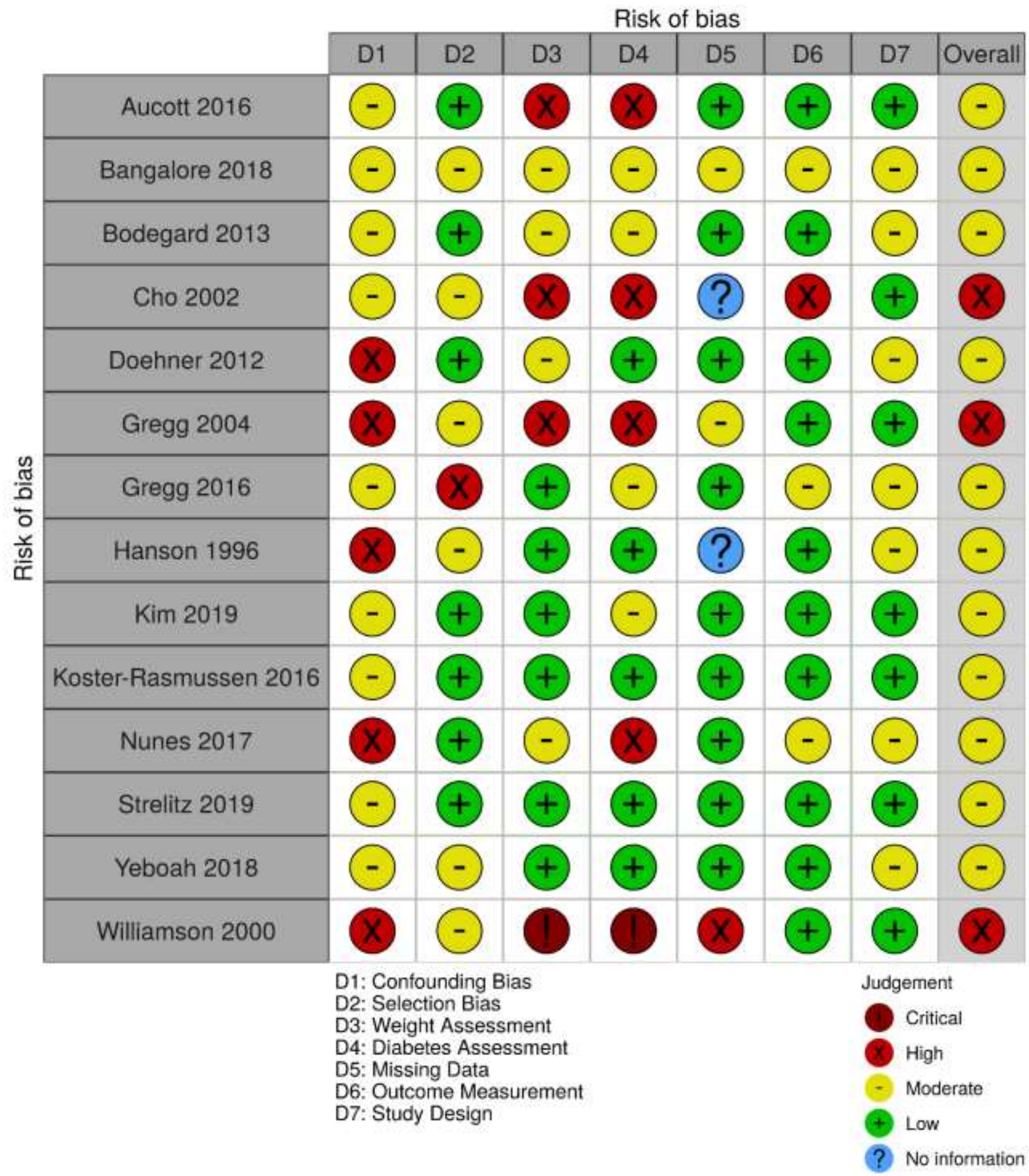

ESM Figure 2. Risk of bias visualisation for intervention trials.

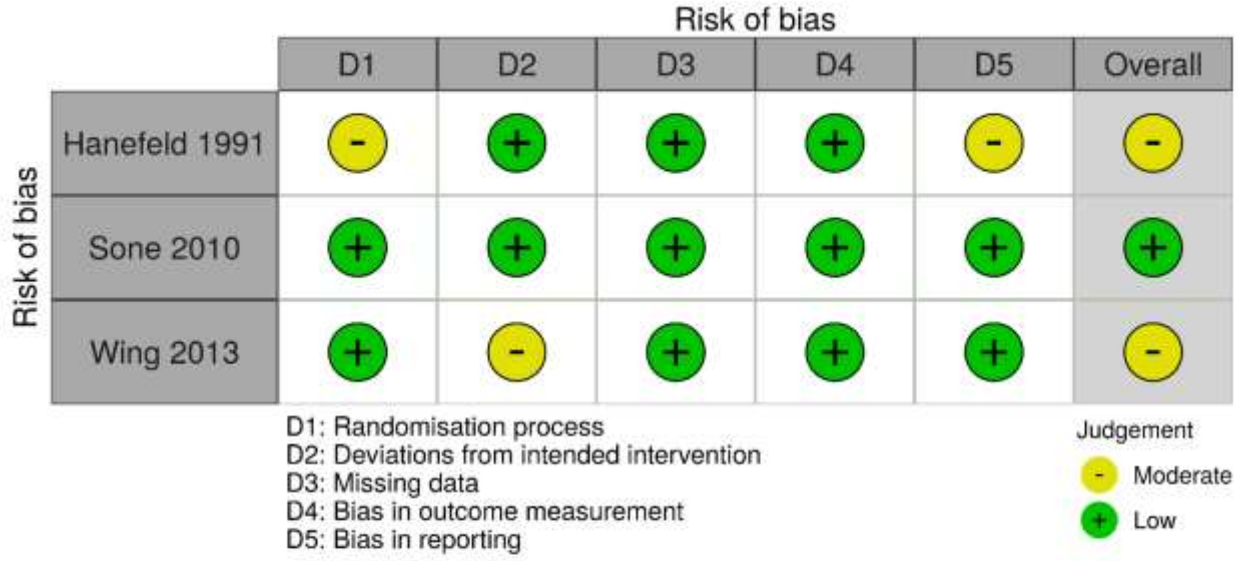

Supplement: Supplementary file 1 — (PDF 260 kb) [file 125_2021_5605_MOESM1_ESM.pdf]
